# Qiling Hushen Formula Ameliorates Type 2 Diabetic Kidney Disease via Gut‐Kidney Axis Restoration and TLR4/NF‐*κ*B/NLRP3 Suppression

**DOI:** 10.1155/jdr/4391252

**Published:** 2026-05-03

**Authors:** Yuxin Wang, Pei Sheng, Xue Wang, Min Lin, Wentao Cai, Shiyao Wei, Yuyun Du, Ming Yan, Lei Wang, Xiaofei An

**Affiliations:** ^1^ School of Traditional Chinese Pharmacy, China Pharmaceutical University, Nanjing, Jiangsu, China, cpu.edu.cn; ^2^ Department of Endocrinology, Affiliated Hospital of Nanjing University of Chinese Medicine, Nanjing, Jiangsu, China, njucm.edu.cn; ^3^ New Drug Screening and Pharmacodynamics Evaluation Center, State Key Laboratory of Natural Medicines, China Pharmaceutical University, Nanjing, Jiangsu, China, cpu.edu.cn

**Keywords:** gut microbiota, gut-kidney axis, intestinal barrier, Qiling Hushen Formula, TLR4/NF-*κ*B/NLRP3 signaling pathway, Type 2 diabetic kidney disease

## Abstract

**Context:**

Qiling Hushen Formula (QLHSF) has been widely used in clinical treatment for Type 2 diabetic kidney disease (T2DKD), yet its mechanism remains unclear.

**Objective:**

This study is aimed at elucidating the protective effects and mechanisms of QLHSF on T2DKD mice.

**Methods:**

Chemical components of QLHSF were analyzed via ultraperformance liquid chromatography coupled with quadrupole time‐of‐flight tandem mass spectrometry (UPLC‐QTOF‐MS/MS). After 6 weeks of QLHSF intervention in C57BL/KSJ db/db mice, urine and serum samples were collected to measure biochemical parameters, inflammatory cytokines, and lipopolysaccharide (LPS) levels. Renal and colonic tissues were sectioned and subjected to histopathological staining. To evaluate the diversity and abundance of the gut microbiota, its composition was determined through 16S rRNA gene sequencing. Expression levels of colonic tight junction proteins, along with key proteins involved in the renal TLR4/NF‐*κ*B signaling pathway and NLRP3 inflammasome activation, were measured.

**Results:**

QLHSF decreased proteinuria and improved renal function in T2DKD mice. Renal pathological injuries and intestinal barrier destruction were also alleviated. Analysis of 16S rRNA sequencing data demonstrated that QLHSF significantly modulated the gut microbiota composition, enriching beneficial bacteria such as *g_Bacteroides*, *g_Lachnospiraceae_NK4A136_group*, *g_Alistipes*, *g_Muribaculum*, *g_Odoribacter*, and *g_Lachnoclostridium*. QLHSF treatment led to a reduction in serum LPS levels and inhibition of the renal TLR4/NF‐*κ*B/NLRP3 signaling pathway, leading to reduced inflammatory cytokine production.

**Conclusions:**

In T2DKD mice, QLHSF ameliorated proteinuria and renal injury through modulation of the gut microbiota, preservation of intestinal barrier integrity, and inhibition of the renal TLR4/NF‐*κ*B/NLRP3 pathway. These findings elucidate the pharmacological mechanism of QLHSF and provide strong experimental support for its clinical application in the treatment of T2DKD.

## 1. Introduction

Diabetic kidney disease (DKD) represents a prevalent microvascular complication arising from diabetes mellitus, with Type 2 diabetic kidney disease (T2DKD) representing a major subtype. As a primary contributor to end‐stage renal disease (ESRD), T2DKD imposes an escalating global health burden. A projection indicated that approximately 550 million individuals will be affected by 2035, driven mainly by the increasing incidence of Type 2 diabetes mellitus [1]. T2DKD is distinguished by key pathological manifestations including heightened proteinuria, reduced glomerular filtration rate, podocyte effacement, mesangial cell hypertrophy, and extracellular matrix accumulation [2–5]. These changes trigger a cascade of renal inflammation, fibrosis, and tissue injury [6]. Currently, pharmacotherapeutic strategies for T2DKD include renin‐angiotensin system (RAS) inhibitors, sodium‐glucose cotransporter‐2 (SGLT2) inhibitors, and mineralocorticoid receptor antagonists (MRAs). Nonetheless, their clinical use is constrained by adverse effects. For instance, treatment with SGLT2 inhibitors has been linked to an elevated incidence of specific adverse effects, including volume depletion, genital and urinary tract infections, euglycemic ketoacidosis, and perineal necrotizing fasciitis [7]. Moreover, despite the therapeutic benefits observed with SGLT2 inhibitors and RAS inhibitors, a significant proportion of patients still progress to chronic kidney disease and ESRD [8]. Therefore, the development of safe and effective therapeutic agents for T2DKD remains imperative.

Qiling Hushen Formula (QLHSF, Approval No. Z20220027001) is an optimized formulation derived from the Xiaoshen Formula (XSF) and is now widely administered clinically for managing T2DKD. QLHSF comprises the following herbs: *Astragalus mongholicus* Bunge, *Poria cocos* (Schw.) Wolf, *Paeonia lactiflora* Pall., *Achyranthes bidentata* Blume, *Imperata cylindrica* (L.) Raeusch., *Lonicera japonica* Thunb., *Luffa acutangula* (L.) Roxb., *Dioscorea oppositifolia* L., *Plantago asiatica* L., and *Cornus officinalis* Siebold & Zucc. According to traditional Chinese medicine theory, these 10 herbs work synergistically to dispel dampness, remove blood stasis, and dredge collaterals. Clinical studies have confirmed QLHSF efficacy in reducing proteinuria in T2DKD patients [9–11]. Previous pharmacological research demonstrated that QLHSF significantly alleviated streptozotocin‐induced renal insufficiency in T2DKD by inhibiting arginase activity and heparinase protein expression [12]. Notably, clinical observations further indicated that QLHSF alleviated T2DKD‐associated symptoms, including anorexia, abdominal distension, and irregular bowel movement, which are related to gastrointestinal dysfunction in traditional Chinese medicine [9]. This is associated with the fact that QLHSF contains traditional Chinese medicines such as *A. mongholicus*, *P. cocos*, and *D. oppositifolia*, which can strengthen the spleen, replenish qi, and remove dampness. Furthermore, a recent systemic study on Qiling Hushen granules identified 49 prototype constituents and 44 metabolites that were significantly distributed in fecal samples and proposed that their therapeutic effects may involve gut microbiota‐mediated modulation of host metabolism [13]. This evidence provided a direct rationale for investigating QLHSF interaction with the gut ecosystem, including the gut microbiota and intestinal barrier.

Gut microbiota comprises trillions of microbes that inhabit the digestive tracts. These microbes engage in intricate relationships with the host, including metabolic processes, immune function, and overall health. It is noteworthy that they were significantly implicated in the pathogenesis of T2DKD [14]. Recent research has underscored the bidirectional communication between the gut microbiota and the kidneys, a concept commonly defined as the gut‐kidney axis [15]. When renal function is impaired, metabolic squanders discharged by the kidneys are moved to the digestive tracts, prompting gut microbiota dysbiosis and the breakdown of the intestinal barrier. In turn, microbiota dysbiosis can influence digestion, which will additionally prompt aggravation in renal tissue [16, 17]. Specifically, gut microbiota dysbiosis compromised intestinal barrier integrity, thereby enabling the translocation of gram‐negative bacterial endotoxins, including lipopolysaccharide (LPS), into the systemic circulation [15, 18]. Gut‐derived LPS served as a key pathological trigger in T2DKD by activating toll‐like receptor 4 (TLR4), driving low‐grade inflammation, renal injury, and fibrosis [19–21]. Moreover, accumulating evidence indicated that traditional Chinese medicine can ameliorate DKD through modulation of the gut‐kidney axis [22, 23]. Consequently, targeting this axis has become a promising therapeutic strategy for traditional Chinese medicine in treating T2DKD.

Although the therapeutic efficacy of QLHSF against T2DKD has been well‐established, its potential mechanisms remain incompletely elucidated. This study was therefore designed to investigate these mechanisms, specifically focusing on the modulation of gut microbiota, intestinal barrier function, and inflammatory signaling. Accordingly, this work was designed to elucidate the mechanism by which QLHSF alleviates T2DKD by restoring the gut‐kidney axis and inhibiting the TLR4/NF‐*κ*B/NLRP3 pathway.

## 2. Materials and Methods

### 2.1. Materials and Reagents

*P. lactiflora*, *A. bidentata*, *L. japonica*, *P. asiatica*, and *D. oppositifolia* were obtained from Anhui Maanshan Jinquan Chinese Medicinal Decoction Pieces Co. (Lot. No: 20200626). *A. mongholicus*, *P. cocos*, and *C. officinalis* were sourced from Guizhou Tongde Pharmaceutical Co. Ltd. (Lot. No: 20200617). *I. cylindrica* was purchased from Anhui Dabieshan Chinese Medicinal Decoction Pieces Co. (Lot. No: 20200825). *L. acutangula* was obtained from Anhui Wansheng Chinese Medicinal Decoction Pieces Co. (Lot. No: 20200806). The plant materials were identified by Prof. Yufeng Zhu of Jiangsu Provincial Hospital of Traditional Chinese Medicine (Nanjing, China). The voucher specimen (No. AXF2020110501) was deposited at the Center of Herbarium, Jiangsu Provincial Hospital of Traditional Chinese Medicine, Nanjing, China (Herbarium Code: QLHS).

Losartan potassium tablets were obtained from Huahai Pharmaceutical Co. Ltd (Zhejiang, China). Biochemical assay kits for total cholesterol (TCHO, Cat# A111‐1‐1), triglyceride (TG, Cat# A110‐2‐1), urea nitrogen (BUN, Cat# C013‐2‐1), glucose (Cat# A154‐2‐1), urinary microalbumin (Cat# E038‐1‐1) and creatinine (CRE, Cat# C011‐2‐1) were acquired from Nanjing Jiancheng Bioengineering Institute (Nanjing, China). Enzyme‐linked immunosorbent assay (ELISA) kits for tumor necrosis factor‐*α* (TNF‐*α*, Cat# KT99985), interleukin‐6 (IL‐6, Cat# KT99854), and interleukin‐1*β* (IL‐1*β*, Cat# KT21178) were provided by Wuhan Mosak Biotechnology Co. Ltd. (Wuhan, China). LPS detection kits (Cat# CSB‐E13066m) were purchased from Wuhan Huamei Bioengineering Co. Ltd (Wuhan, China). Radio immunoprecipitation assay (RIPA) lysis buffer (Cat# WB3100) was obtained from New Cell & Molecular Biotech Co. Ltd (Suzhou, China). Bicinchoninic acid (BCA) protein assay kits (Cat# P0012), protease and phosphatase inhibitor (Cat# P1045) were supplied by Beyotime Biotechnology (Shanghai, China). All antibodies were obtained from commercial sources, as detailed in Table 1.

**Table 1:** Information of antibodies.

Antibody (RRID)	Host species	Vendor	Catalog No.	Application (working dilution)
ZO‐1 (AB_2533938)	Rabbit	Thermo Fisher Scientific	#61‐7300	Western blotting (1: 4000) immunofluorescence (1: 100)
Occludin (AB_2533101)	Mouse	Thermo Fisher Scientific	#33‐1500	Western blotting (1: 4000) immunofluorescence (1: 100)
Claudin 1 (AB_2079881)	Rabbit	Proteintech	#13050‐1‐AP	Western blotting (1: 2000) immunofluorescence (1: 250)
NF‐*κ*B p65 (AB_10859369)	Rabbit	Cell Signaling Technology	#8242	Western blotting (1: 1000)
Phospho‐NF‐*κ*B p65 (AB_331284)	Rabbit	Cell Signaling Technology	#3033	Western blotting (1: 1000)
TLR4 (AB_2798460)	Rabbit	Cell Signaling Technology	#14358	Western blotting (1: 1000)
MyD88 (AB_10547882)	Rabbit	Cell Signaling Technology	#4283	Western blotting (1: 1000)
NLRP3 (AB_2722591)	Rabbit	Cell Signaling Technology	#15101	Western blotting (1: 1000)
Caspase‐1 (AB_2923067)	Rabbit	Cell Signaling Technology	#89332	Western blotting (1: 1000)
ASC (AB_2737351)	Mouse	Santa Cruz Biotechnology	#sc‐514414	Western blotting (1: 1000)
GAPDH (AB_2107436)	Mouse	Proteintech	#60004‐1‐Ig	Western blotting (1: 50000)
Goat anti‐mouse IgG (*H* + *L*) HRP (AB_2722565)	Goat	Proteintech	#SA00001‐1	Western blotting (1:10000)
Goat anti‐rabbit IgG (*H* + *L*) HRP (AB_2722564)	Goat	Proteintech	#SA00001‐2	Western blotting (1:10000)
CoraLite488‐conjugated goat anti‐mouse IgG (*H* + *L*) (AB_2810983)	Goat	Proteintech	#SA00013‐1	Immunofluorescence (1:250)
Goat anti‐rabbit IgG(*H* + *L*) Fluor647‐conjugated (AB_2844801)	Goat	Affinity	#S0013	Immunofluorescence (1:250)

### 2.2. Preparation and Component Analysis of QLHSF

The preparation procedure for QLHSF extract was as follows: 145 g of the mixed herbs was soaked in 700 mL of distilled water for 30 min, followed by a single decoction lasting 30 min. The resulting decoction was then filtered and cooled to room temperature. Ethanol was added to the filtrate to 90% (*v*/*v*) final concentration for precipitation. After 24 h of static sedimentation, the supernatant was collected via vacuum filtration. The solvent was subsequently removed via reduced pressure distillation, after which the residue was dissolved in water. Finally, the aqueous solution was lyophilized to obtain freeze‐dried powder.

To identify chemical components, QLHSF was analyzed by ultraperformance liquid chromatography coupled with quadrupole time‐of‐flight tandem mass spectrometry (UPLC‐QTOF‐MS/MS). Specifically, the freeze‐dried powder was dissolved in 50% acetonitrile and analyzed using an Agilent 1260 system equipped with a Welch Ultimate XB‐C18 column (250 × 4.6 mm, 5 *μ*m). The gradient elution procedure was as follows: 0–5 min, 5% A; 5–15 min, 5%–10% A; 15–17 min, 10% A; 17–27 min, 10%–12% A; 27–29 min, 12%–13% A; 29–31 min, 13% A; 31–36 min, 13%–14% A; 36–48 min, 14%–20% A; 48–55 min, 20%–22% A; 55–80 min, 22%–35% A; 80–90 min, 35%–95% A (A: acetonitrile; B: 0.1% formic acid in water). Operational conditions included: column temperature 25°C, flow rate 1.0 mL/min, and injection volume 10 *μ*L. Mass spectrometric detection was carried out on an Agilent 6530 Q‐TOF spectrometer with an electrospray ionization source. Data were acquired in both positive and negative ionization modes (m/z 20‐2000) to ensure detection of diverse compounds. Key mass spectrometry parameters were set as follows: drying gas (N_2_) flow rate 9.0 L/min, gas temperature 350°C, nebulizer pressure 35 psi, sheath gas temperature 350°C, capillary voltage 4 kV, skimmer voltage 65 V, OCT RFV 750 V, and fragmentor voltage 120 V. Ultimately, data processing was performed using MassHunter 10.0 software.

### 2.3. Animal Experiment

Nine‐week‐old male db/db mice (C57BL/KSJ background) and nondiabetic db/m littermates were purchased from GemPharmatech Co. Ltd. (Nanjing, China, NO. SCXK(SU)2023‐0009). All experimental procedures were conducted following the regulations for the administration of affairs concerning experimental animals (Ministry of Science and Technology, China). This study was reviewed and approved by the Ethics Committee of China Pharmaceutical University on November 21, 2023 (NO. 2023‐11‐011). Animals were housed under a specific–pathogen‐free environment with a maintained temperature of 22^°^C ± 2^°^C, relative humidity of 50%–60% and a 12 h light‐dark cycle. Throughout the study, all mice were allowed free access to food and water.

After 1 week of acclimatization, db/m mice served as a control group, whereas db/db mice were randomly allocated into four groups (*n* = 6 per group): T2DKD group, QLHSF low‐dose group (QLHSF‐L, 9.1 g/kg), QLHSF high‐dose group (QLHSF‐H, 18.2 g/kg), and losartan group (10 mg/kg). QLHSF and losartan were suspended in normal saline. The control group received an equal volume of normal saline daily. All groups received daily intragastric administration for 6 consecutive weeks. The QLHSF dosages were calculated from the clinical dose of QLHSF (145 g/70 kg) using the Meeh‐Rubner equation [24, 25]. Body weights were recorded weekly, and 24‐h urine samples were obtained. At the end of the experiment, the mice were humanely euthanized via cervical dislocation, after which samples (whole blood, kidney, colon, and colonic contents) were collected.

### 2.4. Biochemical Assays of Serum and Urine Samples

Whole blood was allowed to clot at room temperature for 1 h, followed by centrifuging at 3500 rpm for 15 min to isolate serum. Serum levels of TCHO, TG, blood glucose, CRE, and BUN were quantified. Additionally, serum LPS levels were determined using ELISA kits. For urinary analysis, 24‐h urine samples were centrifuged at 2500 rpm for 10 min, and the resulting supernatants were used for further assays. Urinary CRE and microalbumin levels were measured to calculate the urinary albumin‐to‐creatinine ratio (UACR), a key renal function index.

### 2.5. Inflammatory Cytokine Analysis

Inflammatory cytokine analysis was performed in serum, colonic tissues, and renal tissues. Colonic and renal tissues were homogenized in phosphate‐buffered saline to prepare 5% homogenates, which were then centrifuged to collect the supernatants. Concentrations of TNF‐*α*, IL‐6, and IL‐1*β* in both serum and supernatants were measured using corresponding ELISA kits.

### 2.6. Histopathological Examination

Renal tissues were fixed in 4% paraformaldehyde, whereas colonic tissues were fixed in Carnoy′s fluid. Following fixation, all tissues were embedded in paraffin and sectioned (4 *μ*m). The renal sections were stained with hematoxylin‐eosin (H&E) for histopathological damage, periodic acid Schiff (PAS) for glycogen deposition, and Masson′s staining for fibrosis analysis. Colonic sections were analyzed using H&E and Alcian blue‐periodic acid Schiff (AB‐PAS) staining to assess intestinal pathology. All stained sections were imaged using a BX53 microscope (Olympus, Japan). Colonic histology score was evaluated as follows: lymphocyte infiltration (0–4), crypt distortion (0–4), extent of inflammation (0–3), and colon wall distortion (0–3) [26].

### 2.7. Immunofluorescence Staining

Colonic sections were initially fixed in 4% paraformaldehyde (Servicebio, Wuhan, China) and subsequently permeabilized in 0.5% Triton X‐100 (Solarbio, Beijing, China) for 15 min at room temperature. Following a 1‐h blocking step with 5% goat serum (Beyotime, Shanghai, China), the sections were treated overnight at 4°C with primary antibodies against zonula occludens‐1 (ZO‐1), occludin, and claudin 1. Thereafter, the sections were treated with a fluorescein isothiocyanate secondary antibody (Proteintech, Rosemont, United States) for 1 h at room temperature in the dark. Nuclei were counterstained with antifade mounting medium with DAPI (Beyotime, Shanghai, China). Images were performed with a BZ‐X800E microscope.

### 2.8. Western Blotting

Renal and colonic tissues were homogenized in RIPA lysis buffer supplemented with protease and phosphatase inhibitors, followed by centrifugation (12000 g, 10 min) to collect the supernatants. Protein concentrations were then determined with a BCA assay kit. Equal amounts of protein samples were then separated by SDS‐PAGE and transferred onto PVDF membranes (Merck, Darmstadt, Germany). Following a 1.5‐h blocking step with 5% bovine serum albumin at room temperature, the membranes were incubated overnight at 4°C with primary antibodies against ZO‐1, occludin, claudin 1, nuclear factor kappaB p65 (NF‐*κ*B p65), phospho‐NF‐*κ*B p65 (p‐p65), TLR4, myeloid differentiation factor 88 (MyD88), NOD‐like receptor thermal protein domain associated protein 3 (NLRP3), cysteine‐containing aspartate‐specific proteases 1 (caspase1), and apoptosis‐associated speck‐like protein (ASC). Following this, the membranes were incubated with horseradish peroxidase‐conjugated secondary antibodies for 1.5 h at room temperature. Protein bands were visualized by enhanced chemiluminescence (ECL) reagents and quantitatively analyzed with Image J software Version 1.8.0 (National Institutes of Health, United States).

### 2.9. 16S Ribosomal RNA (16S rRNA) Gene Sequencing

Colonic contents were immediately snap‐frozen in liquid nitrogen and preserved at −80°C until analysis. Total genomic DNA was extracted using the Magnetic Soil and Stool DNA Kit (Cat#: DP712). DNA purity and concentration were measured, and the V4 hypervariable region of the bacterial 16S rRNA gene was amplified by PCR using the primers 5 ^′^‐GTGCCAGCMGCCGCGGTAA‐3 ^′^ and 5 ^′^‐GGACTACHVGGGTWTCTAAT‐3 ^′^. The PCR products were combined with an equal volume of 1× loading buffer containing SYBR Green and analyzed by electrophoresis on a 2% agarose gel. Purification was performed using a DNA purification kit (Cat#: DP214), followed by library construction using the NEB Next Ultra II FS DNA PCR‐free Library Prep Kit (Cat#: E7430L). The constructed library was quantified using qubit and real‐time PCR. The quantified libraries were pooled and sequenced on an Illumina NovaSeq PE250 platform. For bioinformatics analysis, paired‐end reads from sequencing were first spliced, filtered and removed chimera to obtain effective tags. Amplicon sequence variants (ASVs) were generated using denoising algorithms. Taxonomic annotation and analyses of species composition, alpha diversity and beta diversity were performed. All sequencing services were provided by Novogene Co. Ltd (Beijing, China).

### 2.10. Statistical Analysis

Statistical analysis was carried out using GraphPad Prism 8.0 software (GraphPad, United States). One‐way analysis of variance (ANOVA) was used for comparisons among three or more groups. Results were presented as mean ± SEM, and differences were deemed statistically significant at *p* < 0.05.

## 3. Results

### 3.1. Chemical Composition of QLHSF

**Figure 1:**
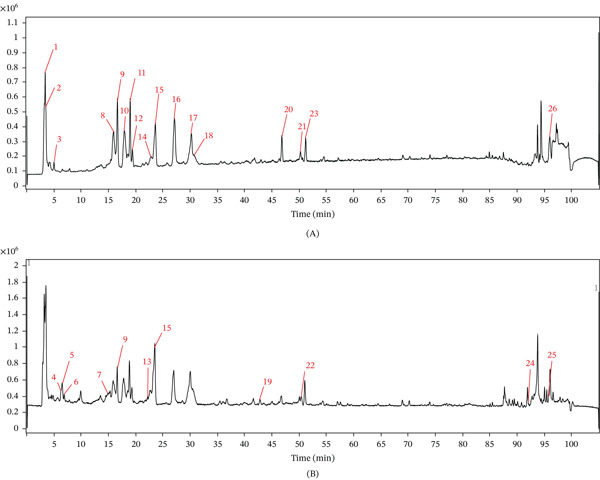
Identification of the components of QLHSF using UPLC‐QTOF‐MS/MS. (A) Positive ion mode. (B) Negative ion mode.

The chemical components of QLHSF were characterized by UPLC‐QTOF‐MS/MS. Through comparative analysis with literature data and querying the Massbank database (https://massbank.eu/MassBank/), 26 chemical components were identified from QLHSF. Detailed information regarding these identified components is provided in Table S1 and Figure 1.

### 3.2. QLHSF Ameliorated Renal Dysfunction and Hyperglycemia in T2DKD Mice

**Figure 2:**
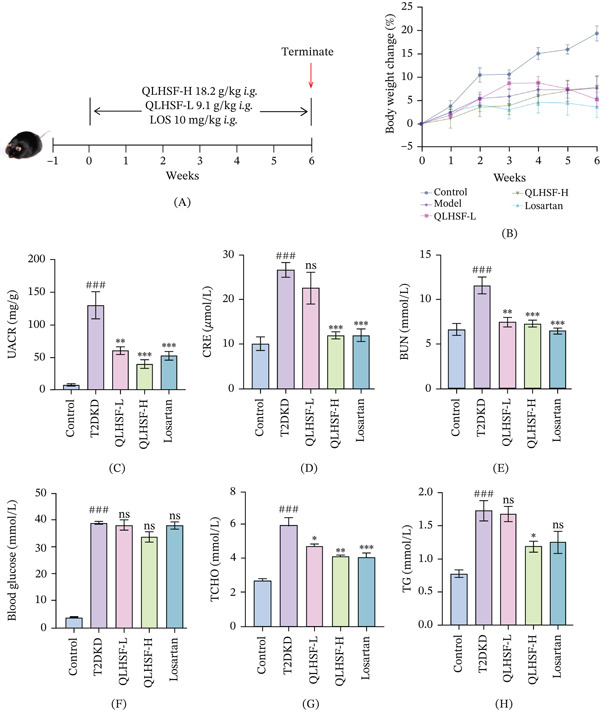
Therapeutic effects of QLHSF on db/db mice. (A) The treatment regimen in this study. (B) The body weight change (%) during the 6‐week intervention period. (C) The levels of UACR. (D) The levels of serum CRE. (E) The levels of serum BUN. (F) The levels of serum blood glucose. (G) The levels of serum TCHO. (H) The levels of serum TG. All data are presented as the mean ± SEM (*n* = 6), ###*p* < 0.001 versus control group; ∗*p* < 0.05, ∗∗*p* < 0.01, ∗∗∗*p* < 0.001 versus T2DKD group.

The treatment protocol is illustrated in Figure 2A. During the intervention period, no significant differences in body weight change were observed between the T2DKD group and the treatment groups (QLHSF‐L, QLHSF‐H, and losartan), indicating that the treatments did not affect body weight in this model (Figure 2B). Compared with the T2DKD group, QLHSF administration significantly reduced UACR and BUN levels in both the low‐dose (*p* < 0.01) and high‐dose (*p* < 0.001) groups (Figure 2C–E). Serum CRE levels were also reduced, but only in the QLHSF‐H group (*p* < 0.001). Serum TCHO levels were decreased in the QLHSF‐L (*p* < 0.05) and QLHSF‐H (*p* < 0.01) groups, whereas serum TG was lowered in the QLHSF‐H group (*p* < 0.05) (Figure 2G,H). Significantly, neither QLHSF nor LOS treatment lowered blood glucose levels (Figure 2F).

### 3.3. QLHSF Alleviated Renal Histopathological Damage and Fibrosis in T2DKD Mice

**Figure 3:**
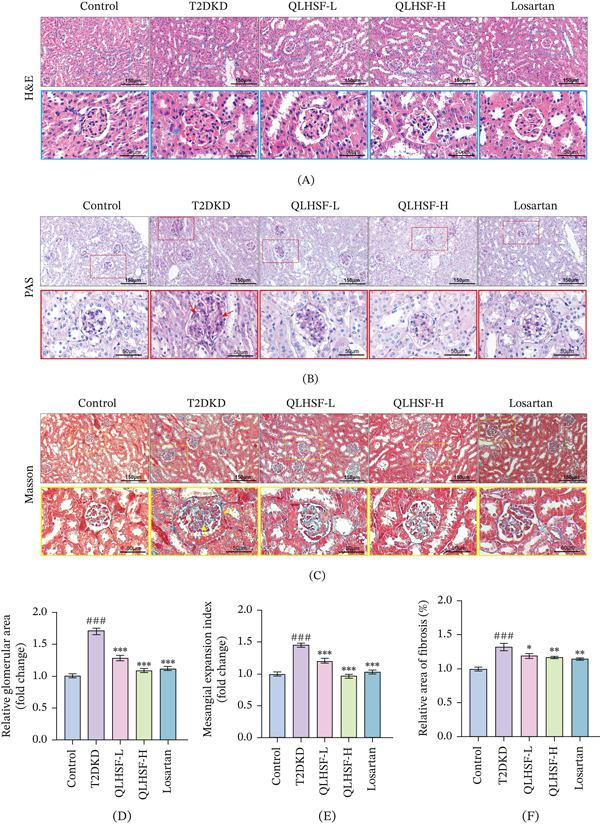
Histopathological analysis of renal tissue. (A–C) Representative H&E, PAS and Masson staining of renal tissue sections. Scale bars: 150 or 50 *μ*m. Arrows indicate glomerular hypertrophy in H&E staining (blue square), glycogen deposition in PAS staining (red square) and collagen fibers in Masson staining (yellow square). (D–F) Semiquantitative assessment of relative glomerular area, mesangial expansion index and positive fibrosis area was conducted with Image J software (v1.8.0). Glomeruli from each section were randomly selected for analysis. All data are presented as the mean ± SEM (*n* = 3), ###*p* < 0.001 versus control group; ∗*p* < 0.05, ∗∗*p* < 0.01, ∗∗∗*p* < 0.001 versus T2DKD group.

Representative images of renal histopathology are shown in Figure 3A–C. H&E staining exhibited marked glomerular hypertrophy in the T2DKD group relative to controls. PAS staining further identified significantly increased glycogen accumulation in the T2DKD group, reflecting increased mesangial expansion and glomerular basement membrane thickening. Additionally, Masson′s staining demonstrated elevated collagen fiber deposition, supporting the presence of renal fibrosis in the T2DKD group. As collectively demonstrated in Figure 3D–F, intervention with either QLHSF significantly alleviated renal damage by mitigating these pathological structural changes and reducing fibrotic deposition.

### 3.4. QLHSF Modulated the Composition of Gut Microbiota in T2DKD Mice

**Figure 4:**
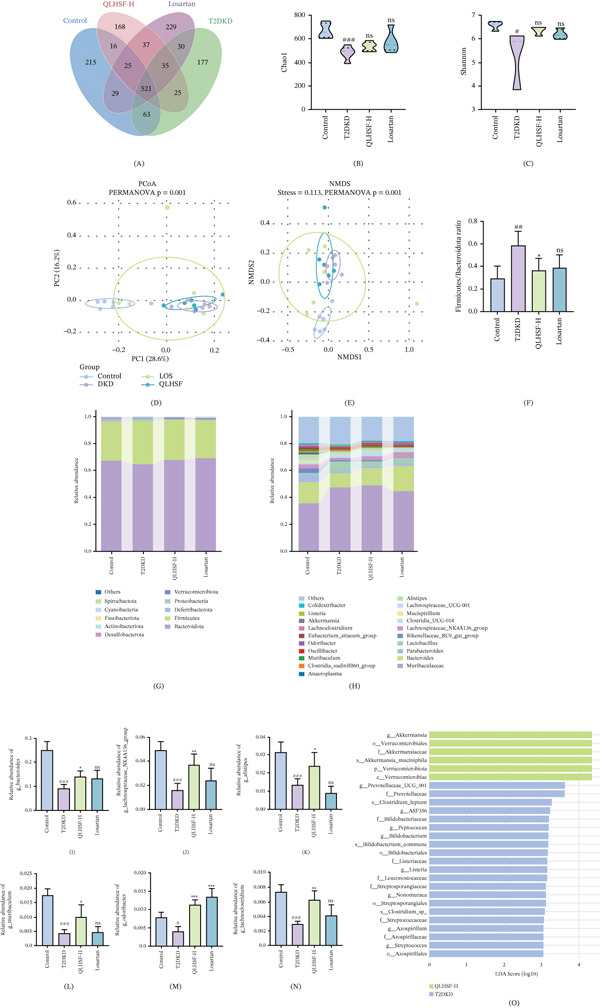
Effects of QLHSF on gut microbiota diversity and composition in db/db mice analyzed by 16S rRNA sequencing. (A) Venn diagram. (B) Chao1 index. (C) Shannon index. (D–E) PCoA and NMDS based on bray curtis. (F) The ratio of Firmicutes to Bacteroidota. (G) Top 10 relative abundance at phylum level. (H) Top 10 relative abundance at genus level. (I–N) Relative abundance of six bacteria with significant changes at genus level: *g_Bacteroides*, *g_Lachnospiraceae_NK4A136_group*, *g_Alistipes*, *g_Muribaculum*, *g_Odoribacter* and *g_Lachnoclostridium*. (O) Taxonomic biomarkers found by LEfSe in T2DKD and QLHSF‐H. The LDA score threshold was 3. All data are presented as the mean ± SEM (*n* = 6), #*p* < 0.05, ##*p* < 0.01, ###*p* < 0.001 versus control group; ∗*p* < 0.05, ∗∗*p* < 0.01, ∗∗∗*p* < 0.001 versus T2DKD group.

To examine the impact of QLHSF on the gut microbiota in T2DKD mice, colonic contents were acquired and subjected to 16S rRNA gene sequencing. As illustrated in Figure 4A, the Venn diagram displayed the common and unique ASVs among the different groups. The control, T2DKD, QLHSF‐H, and losartan groups, respectively, exhibited 215, 177, 168, and 229 unique ASVs. Relative to the control group, alpha diversity, as measured by the Chao1 index (*p* < 0.001) and Shannon index (*p* < 0.05), was reduced in the T2DKD group. Although not statistically significant, QLHSF treatment showed a trend towards increasing both indices (Figure 4B,C). Furthermore, beta diversity was evaluated through principal coordinates analysis (PCoA) and nonmetric multidimensional scaling (NMDS). The results from PCoA and NMDS demonstrated a separation among these groups, suggesting differences in gut microbiota composition (Figure 4D–E).

To investigate the specific alterations in gut microbiota, the relative abundance of microbial species in each group was analyzed. At the phylum level, QLHSF treatment decreased the *Firmicutes* to *Bacteroidota* ratio (*p* < 0.05), a well‐established marker of gut dysbiosis(Figure 4F). Gut microbiota composition at the phylum and genus levels is presented in Figure 4G,H. Among these, six genera exhibited prominent differences in relative abundance between the T2DKD and control groups, including *g_Bacteroides* (*p* < 0.05), *g_Lachnospiraceae_NK4A136_group* (*p* < 0.01), *g_Alistipes* (*p* < 0.05), *g_Muribaculum* (*p* < 0.05), *g_Odoribacter* (*p* < 0.001), and *g_Lachnoclostridium* (*p* < 0.01) (Figure 4I–N). Their relative abundances were reversed by QLHSF‐H treatment. linear discriminant analysis (LDA) effect size (LEfSe) analysis was performed to look for potential biomarkers in the different groups (Figure 4O). The results revealed that *g_Prevotellaceae_UCG_001*, *f_Prevotellaceae*, and *s_Clostridium_leptum* were enriched in the T2DKD group. Conversely, *f_Akkermansiaceae*, *o_Verrucomicrobiales*, and *g_Akkermansia* showed marked enrichment in the QLHSF‐H group.

### 3.5. QLHSF Relieved the Colon Injury in T2DKD Mice

**Figure 5:**
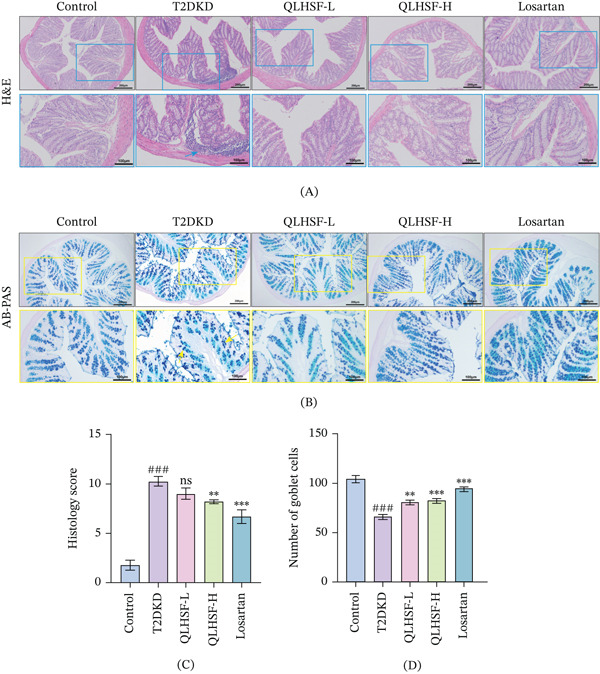
The histopathological analysis of colonic tissue. (A–B) Representative H&E and AB‐PAS staining of colonic tissue sections. Scale bar = 200 or 100 *μ*m. The arrows indicate inflammatory cell infiltration in H&E staining (blue square) and the goblet cell loss in AB‐PAS staining (yellow square) results. (C–D) Colonic tissue sections were subjected to semiquantitative assessment of the histology score and number of goblet cells. All data are presented as the mean ± SEM (*n* = 3), ###*p* < 0.001 vs control group; ∗∗*p* < 0.01, ∗∗∗*p* < 0.001 versus T2DKD group.

Histological examination of colon tissue demonstrated that QLHSF notably reduced inflammatory cell infiltration (Figure 5A). AB‐PAS staining indicated a decrease in mature goblet cells within the T2DKD group, a change that was effectively reversed by QLHSF treatment (Figure 5B). Furthermore, quantitative histopathological assessment confirmed significant improvements in colonic architecture and mucus secretion (Figure 5C,D).

### 3.6. QLHSF Improved the Intestinal Barrier Destruction and Reduced Serum LPS Levels in T2DKD Mice

**Figure 6:**
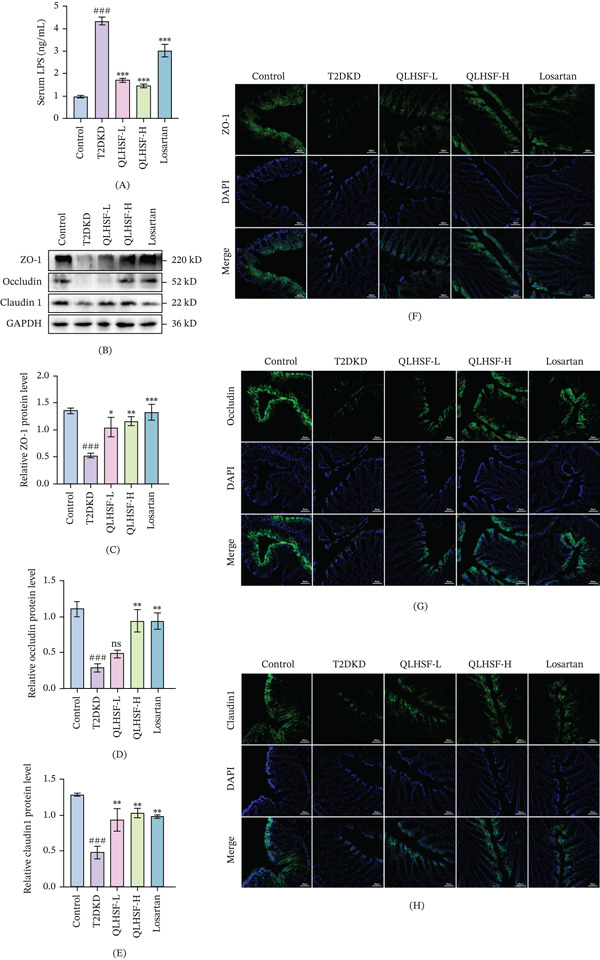
Effect of QLHSF on intestinal barrier function in db/db mice. (A) The level of serum LPS after treatment. (B) Representative western blot results of ZO‐1, occludin and claudin 1 in the colon. (C–E) The quantitative density analysis results of ZO‐1, occludin and claudin 1 in the colon. (F–H) Representative captures of immunofluorescence staining for ZO‐1, occludin and claudin 1 in the colon. All data are presented as the mean ± SEM (*n* = 3–4), ###*p* < 0.001 versus control group; ∗*p* < 0.05, ∗∗*p* < 0.01, ∗∗∗*p* < 0.001 versus T2DKD group.

Serum LPS levels were significantly diminished following QLHSF treatment (*p* < 0.001) (Figure 6A). Moreover, analysis of colonic tight junction proteins revealed that their expression was markedly reduced in the T2DKD group, an effect that was subsequently reversed by QLHSF treatment (Figure 6B–E). Consistent with these findings, immunofluorescence staining results confirmed enhanced expression intensity of these tight junction proteins in the QLHSF group (Figure 6F–H). These results collectively indicated that QLHSF improved intestinal barrier destruction.

### 3.7. QLHSF Improved the Inflammatory Response in T2DKD Mice

**Figure 7:**
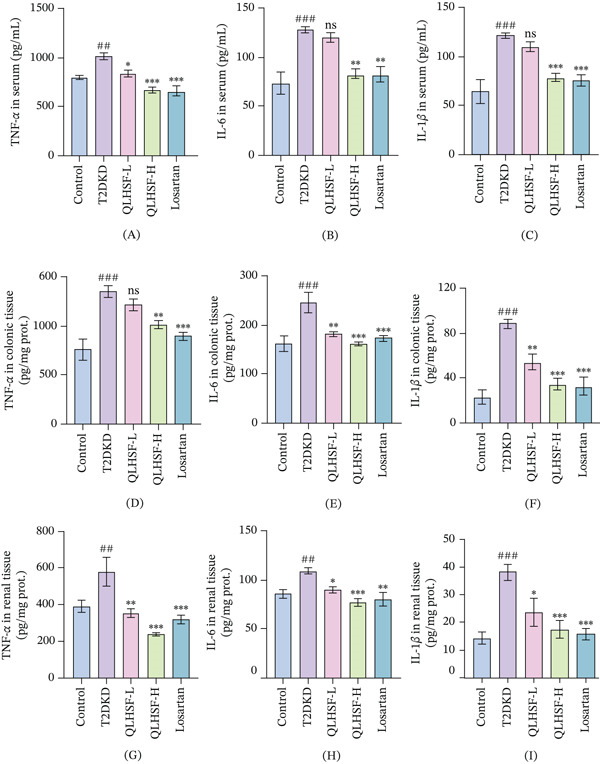
QLHSF alleviated inflammation cytokine levels. (A–C) Expression levels of TNF‐*α*, IL‐6 and IL‐1*β* in serum of db/db mice. (D–F) Expression levels of TNF‐*α*, IL‐6 and IL‐1*β* in colonic tissue of db/db mice. (G–I) Expression levels of TNF‐*α*, IL‐6 and IL‐1*β* in renal tissue of db/db mice. All data are presented as the mean ± SEM (*n* = 6), ##*p* < 0.01, ###*p* < 0.001 versus control group; ∗*p* < 0.05, ∗∗*p* < 0.01, ∗∗∗*p* < 0.001 versus T2DKD group.

As illustrated in Figure 7A–I, the levels of inflammatory cytokines IL‐1*β*, IL‐6, and TNF‐*α* were significantly elevated in serum, colonic tissues, and renal tissues of the T2DKD group relative to controls. Strikingly, treatment with QLHSF effectively reduced the levels of these cytokines. Of particular significance, the QLHSF‐H group demonstrated comparable efficacy in suppressing inflammation to the losartan group.

### 3.8. QLHSF Inhibited the Activation of TLR4/NF‐*κ*B/NLRP3 Signaling Pathway

**Figure 8:**
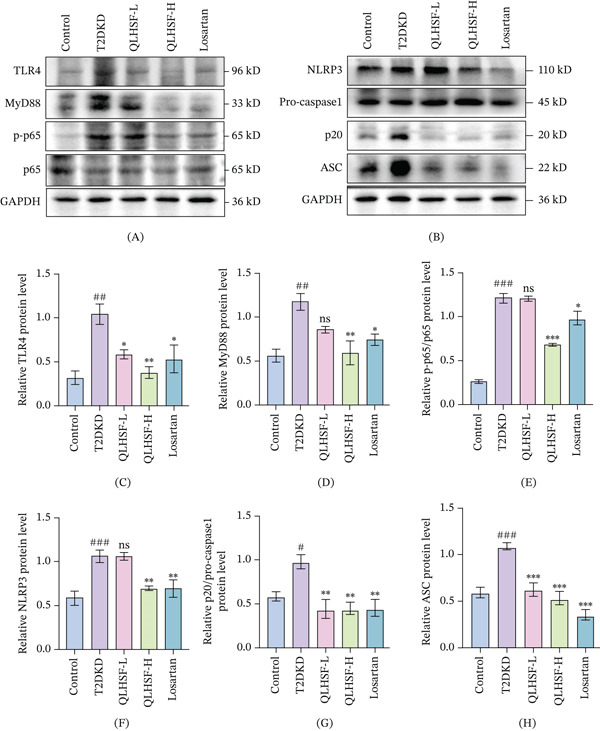
Effects of QLHSF on the TLR4/NF‐*κ*B/NLRP3 signaling pathway. (A–B) Representative western blot bands of proteins associated with the TLR4/NF‐*κ*B pathway (TLR4, MyD88, p‐p65, p65) and NLRP3 inflammasome activation (NLRP3, pro‐caspase‐1, p20 and ASC) in renal tissues. (C–E) Quantification of TLR4, MyD88 and p‐p65/p65 ratio. (F–H) Quantification of NLRP3, pro‐caspase‐1/p20 ratio and ASC. All data are presented as the mean ± SEM (*n* = 3–4), #*p* < 0.05, ##*p* < 0.01, ###*p* < 0.001 versus control group; ∗*p* < 0.05, ∗∗*p* < 0.01, ∗∗∗*p* < 0.001 versus T2DKD group.

After entering systemic circulation, LPS triggers inflammatory cascades by activating TLR4 signaling, ultimately promoting the production of inflammatory cytokines. To investigate this pathway in our model, the levels of proteins related to this pathway were detected by western blot (Figure 8A,B). Relative to controls, the T2DKD group significantly elevated expression levels of TLR4 (*p* < 0.01) and its adaptor molecule MyD88 (*p* < 0.01) in renal tissues. However, QLHSF treatment effectively suppressed their levels (Figure 8C,D). In addition, a marked reduction in the phosphorylation ratio of NF‐*κ*B p65 (p‐p65/p65) (*p* < 0.001) was also evident following QLHSF‐H treatment (Figure 8E). Further analysis revealed that QLHSF also suppressed NLRP3 inflammasome activation, as indicated by decreased expression of NLRP3, ASC, and cleaved caspase‐1 (p20/pro‐caspase‐1) (Figure 8F–H). These data demonstrated that the protective role of QLHSF in the kidney is achieved through suppression of the TLR4/NF‐*κ*B/NLRP3 signaling axis.

### 3.9. Correlation Analysis

**Figure 9:**
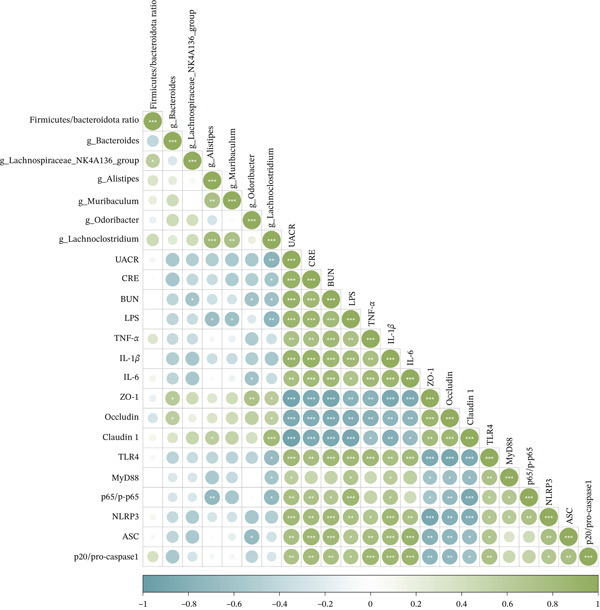
Pearson correlation analysis. Green squares represent positive correlations, blue squares represent negative correlations, and white squares represent no correlations. ∗*p* < 0.05, ∗∗*p* < 0.01, ∗∗∗*p* < 0.001.

The Pearson correlation analysis was performed to examine the associations among gut microbiota, renal function, LPS level, renal inflammation, intestinal integrity, and protein expression (Figure 9). Elevated levels of renal function indices (UACR, CRE, and BUN) were positively associated with the concentration of inflammatory cytokines (IL‐1*β*, IL‐6, and TNF‐*α*), LPS level, and the expression of proteins in the TLR4/NF‐*κ*B/NLRP3 pathway. Conversely, the intestinal barrier (ZO‐1, occludin, and claudin 1) demonstrated negative correlations with these renal function indices. Of particular note, LPS level was strongly correlated with renal inflammatory cytokines and TLR4/NF‐*κ*B/NLRP3 pathway proteins while exhibiting a negative correlation with intestinal tight junction protein expression. Furthermore, significant associations were observed between tight junction protein expression and the abundance of specific gut microbes, including *g_Bacteroides*, *g_Alistipes*, *g_Odoribacter*, and *g_Lachnoclostridium*. These findings suggested a potential mechanistic link underlying the gut‐kidney axis.

## 4. Discussion

**Figure 10:**
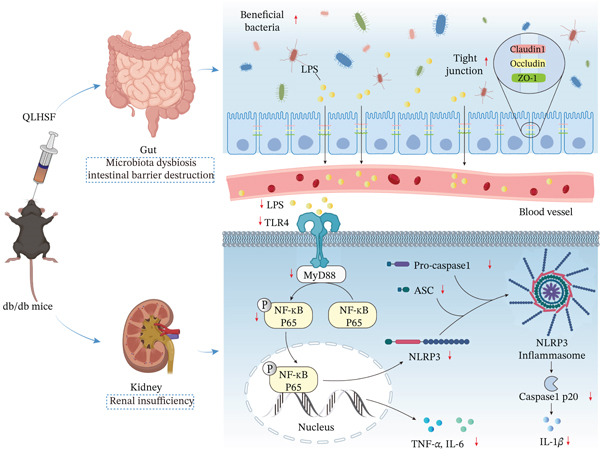
Schematic diagram of QLHSF ameliorates T2DKD in db/db mice via gut‐kidney axis restoration and TLR4/NF‐*κ*B/NLRP3 inflammatory pathway suppression.

The present study was designed to explore therapeutic effects and potential mechanisms of QLHSF in T2DKD mice. The results showed that QLHSF significantly alleviated proteinuria, renal injury, and dyslipidemia. Losartan was included as a positive control given its established role in reducing proteinuria and preserving renal function, thereby providing a clinically relevant benchmark for renoprotection. Importantly, the beneficial effects of QLHSF appeared to be mediated through modulation of the gut‐kidney axis, as evidenced by the amelioration of gut microbiota dysbiosis, enhancement of intestinal barrier integrity, and inhibition of the renal TLR4/NF‐*κ*B/NLRP3 inflammatory pathway (Figure 10). To our knowledge, this is the first study to demonstrate that QLHSF ameliorated T2DKD by modulating the functional crosstalk between the gut and kidney, offering novel insights for its clinical application.

Gut microbiota has been implicated in the development and progression of T2DKD, and an increasing number of traditional Chinese medicines have been shown to alleviate T2DKD by regulating the gut microbiota [27]. To elucidate the microbiota‐dependent mechanism through which QLHSF ameliorated T2DKD, 16S rRNA gene sequencing was performed. Our data demonstrated gut microbiota disruption in T2DKD mice and its restoration by QLHSF treatment. Notably, at the genus level, the effects of QLHSF included enriching bacteria linked to anti‐inflammatory factors (*g_Bacteroides* and *g_Alistipes*) and short‐chain fatty acid (SCFA)‐producing bacteria (*g_Odoribacter*, *g_Lachnospiraceae_NK4A136_group* and *g_Lachnoclostridium*) [28–30]. Given that SCFAs have been shown to regulate lipid metabolism, QLHSF‐mediated enrichment of SCFA‐producing bacteria may enhance SCFA production, thereby contributing to the improved lipid metabolism observed in T2DKD mice [31]. Concurrently, QLHSF treatment increased the relative abundance of *g_Muribaculum*, a taxon whose reduction is associated with inflammation, dyslipidemia and poor glucose tolerance [32]. The restoration of this metabolically relevant bacterium, alongside the amelioration of systemic dyslipidemia, suggests that QLHSF may modulate host metabolism partly through direct microbial regulation. However, whether this is a primary effect or secondary to broader metabolic improvement requires further validation.

These beneficial changes in the microbiota, especially the increase in SCFA‐producing bacteria, are crucial for reinforcing intestinal barrier function [33]. This barrier is preserved by multiprotein complexes termed tight junctions, which consist of transmembrane proteins (e.g., claudin, occludin) and cytoplasmic scaffolding proteins such as ZO‐1 [34, 35]. However, impairment of this barrier directly induced systemic and intestinal inflammation and was a critical driver in the development of multiple disorders, including T2DKD [35, 36]. A key consequence of a compromised barrier is the translocation of microbial products, notably LPS. Under normal conditions, LPS is scarcely detectable in serum, but when the intestinal barrier was compromised, LPS translocated into circulation, causing a rise in its serum levels [37]. Supporting this, our study in the db/db mice confirmed significant colonic tissue damage along with a marked downregulation of tight junction protein expression, indicating intestinal barrier destruction. Importantly, QLHSF treatment restored intestinal barrier function and attenuated serum LPS levels.

In T2DKD conditions, gut‐derived LPS potently activates TLR4, triggering MyD88‐dependent NF‐*κ*B signaling that promoted NLRP3 inflammasome activation and inflammatory cytokines production [38, 39]. Consequently, this inflammatory cascade was known to drive disease progression in T2DKD [40]. Consistent with this, our results demonstrated that QLHSF treatment significantly suppressed key mediators of this pathway, including TLR4, MyD88, NLRP3, ASC, and caspase‐1 activation, while also inhibiting p65 phosphorylation. These inhibitory effects directly corresponded to reduced inflammation in T2DKD.

Correlation analysis further strengthened the association between gut microbiota modulation and therapeutic effects. The relative abundance of beneficial bacteria enriched by QLHSF was positively correlated with intestinal tight junction protein expression but negatively correlated with renal function indices, serum LPS levels, and inflammatory cytokines. These correlations underscore the critical role of gut microbiota composition in preserving intestinal barrier integrity and restraining LPS‐induced renal inflammation via the gut‐kidney axis.

Nevertheless, several limitations of the present study must be considered. Although our findings demonstrated modulatory effects of QLHSF on gut microbiota, future investigations should incorporate fecal microbiota transplantation combined with multiomics approaches to establish mechanistic causality. In addition, the dose of QLHSF requires further optimization. These refinements would not only better elucidate microbiota‐mediated mechanisms underlying therapeutic effects of QLHSF on T2DKD but also facilitate its clinical translation.

## 5. Conclusion

In summary, QLHSF treatment ameliorated proteinuria and renal injury in T2DKD mice by modulating gut microbiota, enhancing intestinal barrier integrity and suppressing the LPS‐triggered TLR4/NF‐*κ*B/NLRP3 inflammatory pathway. These findings provide evidence for the pharmacological mechanism of QLHSF and offer strong experimental support for the clinical application of QLHSF in T2DKD treatment.

**Nomenclature**
16S rRNA: 16S ribosomal RNA
AB‐PAS: Alcian blue‐periodic acid Schiff
ASC: apoptosis‐associated speck‐like protein
ASVs: amplicon sequence variants
BCA: bicinchoninic acid
BUN: urea nitrogen
Caspase1: cysteine‐containing aspartate‐specific proteases 1
CRE: creatinine
DKD: diabetic kidney disease
ECL: enhanced chemiluminescence
ELISA: enzyme‐linked immunosorbent assay
ESRD: end‐stage renal disease
H&E: hematoxylin‐eosin
IL‐1*β*
: interleukin‐1*β*
IL‐6: interleukin‐6
LEfSe: linear discriminant analysis effect size
LPS: lipopolysaccharide
MRAs: mineralocorticoid receptor antagonists
MyD88: myeloid differentiation factor 88
NF‐*κ*B p65: nuclear factor kappaB p65
NLRP3: NOD‐like receptor thermal protein domain associated protein 3
NMDS: nonmetric multidimensional scaling
PAS: periodic acid Schiff
PCoA: principal coordinates analysis
PCR: polymerase chain reaction
P‐p65: phospho‐NF‐*κ*B p65
QLHSF: Qiling Hushen Formula
RAS: renin‐angiotensin system
RIPA: radio immunoprecipitation assay
SCFA: short‐chain fatty acid
SGLT2: sodium‐glucose cotransporter‐2
T2DKD: Type 2 diabetic kidney disease
TCHO: total cholesterol
TG: triglyceride
TLR4: toll‐like receptor 4
TNF‐*α*
: tumor necrosis factor‐*α*
UACR: albumin‐to‐creatinine ratio
UPLC‐QTOF‐MS/MS: ultra performance liquid chromatography coupled with quadrupole time of flight tandem mass spectrometry
XSF: Xiaoshen Formula
ZO‐1: zonula occludens‐1

## Author Contributions

Y.W.: investigation, data curation, visualization, formal analysis, writing—original draft. P.S.: visualization, formal analysis, writing—original draft. X.W.: investigation, data curation, formal analysis, writing—original draft. M.L.: conceptualization, investigation, data curation, writing—original draft. W.C.: investigation, formal analysis, writing—original draft. S.W.: writing—review and editing. Y.D.: writing—review and editing. M.Y.: methodology, writing—review and editing. L.W.: methodology, writing—review and editing. X.A.: funding acquisition, supervision, writing—review and editing. Y.W., P.S., and X.W. have contributed to the work equally and should be regarded as co‐first authors.

## Funding

This study was supported by the National Natural Science Foundation of China (No.82374386).

## Ethics Statement

This study was reviewed and approved by the Ethics Committee of China Pharmaceutical University on November 21, 2023 (NO. 2023‐11‐011).

## Conflicts of Interest

The authors declare no conflicts of interest.

## Supporting information


**Supporting Information** Additional supporting information can be found online in the Supporting Information section. Table S1 Chemical components of QLHSF.

## Data Availability

The data that support the findings of this study are available from the corresponding authors upon reasonable request.
