# Supplementary material for: Qiling Hushen Formula Ameliorates Type 2 Diabetic Kidney Disease via Gut‐Kidney Axis Restoration and TLR4/NF‐κB/NLRP3 Suppression
Source: J Diabetes Res. 2026 May 3;2026:4391252. doi: 10.1155/jdr/4391252 (PMC13136592; doi:10.1155/jdr/4391252)
Supplement: Supplementary file 1 — Supporting Information Additional supporting information can be found online in the Supporting Information section. Table S1 Chemical components of QLHSF. [file JDR-2026-4391252-s001.docx]

Supplementary Material

# Calculation of QLHSF Doses for Mice

The clinical dose of QLHSF for adults (70 kg body weight) is 145 g per day, which is approximately 2 g/kg (145 g/70 kg).

To convert this human equivalent dose to the appropriate dose for mice, the body surface area normalization method was applied, using a conversion factor (Kₘ) of 9.1 for converting a human dose (mg/kg) to a mouse dose (mg/kg). The calculation is as follows:

Mouse Dose (mg/kg) = Human Dose (mg/kg) × Kₘ

Mouse Dose = 2 g/kg × 9.1 = 18.2 g/kg

This dose was designated as the high dose (QLHSF-H). The low dose (QLHSF-L, 9.1 g/kg) was set at half of the high dose to evaluate a potential dose-response relationship.

# Table S1 Chemical components of QLHSF.

| No. | Retention time(min) | Compound | Formula | Adduct ion | m/z value | MS/MS |  |
| --- | --- | --- | --- | --- | --- | --- | --- |
| 1 | 3.41 | Malic acid | C_4_H_6_O_5_ | [M-H]^-^ | 133.0141 | 71.0141;115.0031;133.0136 | |
| 2 | 3.55 | Citric acid | C_6_H_8_O_7_ | [M-H]^-^ | 191.0197 | 41.0049;57.0349;59.013;87.009;111.0077;111.0966;112.0118;129.0173;130.9981 | |
| 3 | 5.03 | Gallic Acid | C_7_H_6_O_5_ | [M-H]^-^ | 169.0143 | 51.02042;67.02;69.0333;79.0187;81.0352;97.0285;107.0147;180.0199;124.0161;125.0232;125.1173;126.0277;127.0312;169.0138 | |
| 4 | 6.27 | Maltol | C_6_H_6_O_3_ | [M+H]^+^ | 127.0401 | 43.0196;81.0343 | |
| 5 | 6.50 | 5-Hydroxymethylfurfural | C_6_H_6_O_3_ | [M+H]^+^ | 127.0398 | 41.0395;69.0347;81.0346;109.0291 | |
| 6 | 6.88 | Phenylalanine | C_9_H_11_NO_2_ | [M+H]^+^ | 166.0864 | 65.0407;77.04;79.055;80.0501;91.0554;93.0686;102.0482;103.0552;104.061;120.0781 | |
| 7 | 14.91 | Morroniside | C_17_H_26_O_11_ | [M+Na]^+^ | 429.1369 | 161.0169;179.0338 | |
| 8 | 15.89 | Neochlorogenic acid | C_16_H_18_O_9_ | [M-H]^-^ | 353.0888 | 127.0398;135.0444;161.0236;173.0441;191.0559;192.0588;193.0609 | |
| 9 | 16.63 | Chlorogenic Acid | C_16_H_18_O_9_ | [M+H]^+^ | 355.1026 | 89.0397;107.0498;117.0344;118.0367;135.0447;136.0469;145.0288;146.0316;163.0393;164.0424 | |
|  |  |  |  | [M-H]^-^ | 353.0884 | 59.0146;71.0139;85.0296;93.0351;109.0287;111.045;127.0398;133.0293;134.0383;135.0445;137.0235;161.0237;171.0294;173.0425;191.056;192.0589;193.0611 | |
| 10 | 17.90 | Secologanoside | C_16_H_22_O_11_ | [M-H]^-^ | 389.1094 | 59.0144;69.035;71.0147;85.0296;89.0248;101.0235;113.0208;121.0658;139.0042 | |
| 11 | 19.00 | Loganin | C_17_H_26_O_10_ | [M-H]^-^ | 389.1097 | 59.0145;101.0247;102.0288;106.0425;166.0603;209.0453;389.1094 | |
| 12 | 19.43 | Swertiamarin | C_16_H_22_O_10_ | [M-H]^-^ | 373.1147 | 55.0187;59.0144;60.0177;67.0181;69.0349;71.0142;73.031;85.0304;89.0249;93.0349;94.0344;95.0119;97.0296;98.0325;101.0244;105.0354;106.0387;113.0235;114.0298;119.035;120.0372;121.0648;123.0449;141.0193;149.0612;150.0644;161.0449;167.0681;179.0521;193.0507;373.1152;375.1238 | |
| 13 | 22.14 | Polyporenic acid C | C_31_H_46_O_4_ | [M+Na]^+^ | 505.203 | 43.0197;69.0347;77.0413;79.0583;85.0297;95.0518;97.0639;99.0485;103.0545;107.0498;109.0649;119.0677;123.0574;128.0508;130.0657;131.0724;133.0136;137.0621;147.0553;149.077;154.0509;155.0675;156.0614;165.0538;166.0584;180.0606;223.1057;281.1142;307.1299 | |
| 14 | 22.75 | 7-Epi-loganin | C_17_H_26_O_10_ | [M+COOH]^-^ | 435.1513 | 127.0405;101.0229 | |
| 15 | 23.41 | Albiflorin | C_23_H_28_O_11_ | [M+H]^+^ | 481.1717 | 77.04;151.0751;152.0785;161.0596;180.0767;197.0812;198.0848;301.1069;319.1174;320.1203;481.1664 | |
|  |  |  |  | [M+HCOO]^-^ | 525.1614 | 77.0402;121.029;151.0718;479.1567 | |
| 16 | 27.13 | Peaoniflorin | C_23_H_28_O_11_ | [M+COOH]^-^ | 525.1629 | 71.0137;77.0392;89.0243;101.0244;113.0239;121.0292;122.0325;123.0437;161.0448;165.055;166.0593;177.0553;309.0968;327.1085;328.1119;431.1335;449.1452;450.1484;451.1524;479.1565;480.1608;525.1582 | |
| 17 | 30.18 | Secoxyloganin | C_17_H_24_O_11_ | [M-H]^-^ | 403.1253 | 101.0242;121.0294;123.0438;127.0394;149.027;165.0561;191.0348;233.0589;371.1002 | |
| 18 | 30.71 | Secologanin | C_17_H_24_O_10_ | [M+COOH]^-^ | 433.1357 | 101.0236;119.0325;123.0455;155.0342;179.0528 | |
| 19 | 42.78 | Poricoic acid B | C_30_H_44_O_5_ | [M+H]^+^ | 485.1998 | 135.0048;169.1317;485.3843 | |
| 20 | 46.80 | Isochlorogenic acid B | C_25_H_24_O_12_ | [M-H]^-^ | 515.1197 | 111.0433;135.0442;155.0354;161.0223;173.0445;179.0338;191.0555;353.0865 | |
| 21 | 50.28 | Cornuside | C_24_H_30_O_14_ | [M-H]^-^ | 541.1576 | 125.025;169.0142;309.0639;347.0753;541.1569 | |
| 22 | 50.36 | Calycosin-7-O-Glc-6-O-Mal | C_25_H_24_O_13_ | [M+H]^+^ | 533.1295 | 137.0233;197.0609;213.0553;225.0557;253.0492;270.053 | |
| 23 | 51.21 | Isochlorogenic acid C | C_25_H_24_O_12_ | [M-H]^-^ | 515.1212 | 93.0349;111.0433;135.0442;155.0354;173.0445;179.0338;191.055;203.0359;299.0524;335.0767;353.0865 | |
| 24 | 91.89 | α-Linolenic Acid | C_18_H_30_O_2_ | [M+H]^+^ | 279.1603 | 57.0711;65.0406;149.0238;149.1246;150.027;205.0883 | |
| 25 | 95.75 | Flindersine | C_14_H_13_NO_2_ | [M+H]^+^ | 228.1966 | 107.0858;113.0942;117.0748;120.0894;132.0864;174.1348;228.1239 | |
| 26 | 95.93 | Pachymic acid | C_33_H_52_O_5_ | [M-H]^-^ | 527.3186 | 117.0357;175.1131 | |
